# The feasibility and benefit of a brief psychosocial intervention in addition to early palliative care in patients with advanced cancer to reduce depressive symptoms: a pilot randomized controlled clinical trial

**DOI:** 10.1186/s12885-017-3560-6

**Published:** 2017-08-23

**Authors:** Thamires Monteiro do Carmo, Bianca Sakamoto Ribeiro Paiva, Cleyton Zanardo de Oliveira, Maria Salete de Angelis Nascimento, Carlos Eduardo Paiva

**Affiliations:** 1 Health-Related Quality of Life Research Group (GPQual), Barretos Cancer Hospital, Barretos, SP Brazil; 2 Center for Research Support (NAP), Barretos Cancer Hospital, Barretos, SP Brazil; 3 Palliative Care Department, Barretos Cancer Hospital, Barretos, SP Brazil; 4 Department of Clinical Oncology, Barretos Cancer Hospital, Barretos, SP Brazil; 5 Departamento de Oncologia Clínica, Divisão de Mama e Ginecologia, Rua Antenor Duarte Vilella, 1331, Bairro Dr Paulo Prata, Barretos, SP CEP: 14784-400 Brazil

**Keywords:** Neoplasms, Palliative care, Cognitive therapy, Depression, Anxiety

## Abstract

**Background:**

The aim of this study was to assess the feasibility and potential benefit of a brief psychosocial intervention based on cognitive-behavioral therapy performed in addition to early palliative care (PC) in the reduction of depressive symptoms among patients with advanced cancer.

**Methods:**

An open-label randomized phase II clinical trial with two intervention arms and one control group. Patients with advanced cancer starting palliative chemotherapy and who met the selection criteria were included. The participants were randomly allocated to three arms: arm A, five weekly sessions of psychosocial intervention combined with early PC; arm B, early PC only; and arm C, standard cancer treatment. Feasibility was investigated by calculating rates (%) of inclusion, attrition, and contamination (% of patients from Arm C that received PC). Scores of depression (primary aim), anxiety, and quality of life were measured at baseline and 45, 90, 120, and 180 days after randomization.

**Results:**

From the total of 613 screened patients (10.3% inclusion rate), 19, 22, and 22 patients were allocated to arms A, B, and C, respectively. Contamination and attrition rates (180 days) were 31.8% and 38.0%, respectively. No interaction between the arms and treatments were found. Regarding effect sizes, there was a moderate benefit in arm A over arms B and C in emotional functioning (−0.66 and −0.61, respectively) but a negative effect of arm A over arm C in depression (−0.74).

**Conclusions:**

Future studies to be conducted with this population group need to revise the eligibility criteria and make them less restrictive. In addition, the need for arm C is questioned due to high contamination rate. The designed psychosocial intervention was not able to reduce depressive symptoms when combined with early PC. Further studies are warrant to evaluate the intervention on-demand and in subgroups of high risk of anxiety/depression.

**Trial registration:**

Clinical Trials identifier NCT02133274. Registered May 6, 2014.

**Electronic supplementary material:**

The online version of this article (doi:10.1186/s12885-017-3560-6) contains supplementary material, which is available to authorized users.

## Background

There has been much discussion in recent years about the integration of palliative care (PC) in oncology [1, 2]. Important clinical trials [3–6] that demonstrate the benefits of the early integration of PC in oncology have been published. Such studies show improvement in patients’ quality of life (QOL) [3–5], lower rates of depressive symptoms [3, 5], less aggressive end-of-life care [3, 6], greater patients’ [4] and caregivers’ [7] satisfaction with the care received. Although the aforementioned evidence, PC continues to be offered late even at comprehensive oncological centers [8].

Countless barriers hinder the access of patients with advanced cancer to PC [9, 10]. Among such barriers, those related to the stigma associated with PC by patients themselves seem relevant in Brazil; many patients believe that PC is merely “a place to die” [11]. At earlier stages of diseases, when patients are functionally fitter, many of them do not accept early referral to PC. In addition, the rate of absenteeism among PC consultations is around 25% in our hospital (personal communication). The cognitive-behavioral therapy (CBT) aims to enable individuals to identify and modify their distorted or dysfunctional automatic thoughts [12]. A CBT-based intervention, including psychoeducation, would be useful to educate patients about PC, reducing stigmatization and facilitating the early transition to PC.

The Pre-Palliative Emotional Care (PREPArE) trial was designed to develop a strategy to prepare patients before their first visit to a PC service. Our hypothesis was that among non-depressed patients with advanced cancer starting first-line palliative chemotherapy, the early provision of PC would be associated with lower rates of depressive symptoms compared to standard cancer treatment. The inclusion of a brief psychosocial intervention based on CBT [12] would be feasible and help reduce the rates of depressive symptoms when systematically combined with early PC.

In the present article, we present the impact of interventions on patients’ emotional domain over time and in greater detail on intervention day 90 measured using several assessment instruments.

## Methods

### Ethics approval and consent to participate

This study was developed according to the standards of the National Health Council Resolution number 466/12 and the guidelines of the Declaration of Helsinki. The study protocol was initially approved in June 2014 by the Research Ethics Committee of the Barretos Cancer Hospital (CEP/HCB n° 699/014) and all participants signed a free and informed consent form.

### Design

A single-center, open-label, randomized phase II clinical trial with two intervention arms and one control group was used (Clinical Trials no. NCT02133274).

The participants were randomized in a 1:1:1 ratio into arms A, B, and C: A (intervention) – a brief CBT-based psychosocial intervention + early PC combined with standard cancer treatment; B (intervention) – early PC combined with standard cancer treatment; and C (control) – standard cancer treatment.

Randomization was performed in blocks of six participants and was stratified according to the primary tumor site. One trained member of the Center for Research Support at Barretos Cancer Hospital (BCH; Barretos, SP, Brazil) who did not participate in data collection or statistical analysis was charged with the randomization, for which purpose random number tables were used.

The interventions were performed by two psychologists specifically trained in the study procedures. Data collection was performed by research coordinators from the Center for Research Support at BCH.

### Eligibility criteria

#### Inclusion criteria

(1) Age ≥ 18 and <75 years old; (2) awareness of the cancer diagnosis; (3) starting first-line palliative chemotherapy; (4) Eastern Cooperative Oncology Group (ECOG-PS) ≤2; (5) life expectancy ≥6 to <24 months (according to the clinical oncologist’s opinion); (6) exhibiting one of the following metastatic or recurrent incurable cancers: breast cancer, platinum-resistant ovarian cancer, cervical cancer, endometrial cancer, neck and head cancer (after radiotherapy failure), castration-resistant prostate cancer, genitourinary cancer (bladder, kidney, testicular, penile), lung cancer, gastrointestinal cancer (colorectal, pancreas, liver, gastric, esophageal, gallbladder).

#### Exclusion criteria

(1) Being under psychological treatment for any psychological disorder; (2) being under pharmacological treatment with antidepressants for depressive disorders and/or anxiety; (3) being under regular follow up at the PC unit; (4) the need for immediate consultation at the PC unit according to the attending physician’s opinion; (5) cognitive or attention deficits impairing subjects from responding to questionnaires or understanding the study (according to the researcher’s opinion); (6) a current of previous diagnosis of any of the following mental disorders: substance-related disorders; schizophrenia and other psychotic disorders, mood disorders (depressive disorders, bipolar disorders); anxiety disorders; dissociative disorders; personality disorders; and/or history of suicide attempt – to be detected by questioning subjects; no confirmatory instrument was applied; (7) cancer with single resected metastasis; (8) the presence of any comorbidity likely to hinder subjects from participating in the study according to the researcher’s opinion; and (9) unavailability (for any reason) to perform the required hospital visits.

### Care as usual

The Palliative Care Unit of BCH offers an outpatient clinic and an inpatient ward with 52 beds that are dedicated to cancer patients who are receiving PC. The Palliative Care Unit includes a medical team and a comprehensive multidisciplinary team. Patients with advanced cancers are referred to PC as per attending oncologists’ decisions; there is no standard protocol guiding referrals.

### Study interventions

The psychosocial intervention was based on CBT techniques; the protocol was developed based on the session structure method formulated by previous researchers [13, 14]. The protocol consisted of five weekly individual sessions performed in a room fit to receive the participants. In short, the sessions were meant to provide psychoeducation on the patients’ current clinical condition and the aims of PC, the functioning of anxiety, and techniques to manage symptoms, discuss depressive symptoms, and techniques for the detection and questioning of automatic thoughts as well as their influence and essential role in the triggering of emotions and behaviors. The first PC appointment were planned to occur after the first two sessions of the psychosocial and educational intervention for participants who are allocated to Arm A. Supplementary Table S1 details the structured psychosocial and educational intervention [see Additional file 1].

Regarding early PC intervention, the participants randomly allocated to arms A and B were systematically scheduled to visit the PC unit in which they were assessed by PC physicians every 3 ± 1 weeks; the first visit was scheduled to occur two or 3 weeks after randomization. Six PC doctors have received training and participated in this study. The appointments followed a standard care protocol, which was duly recorded in medical records using a standardized protocol for filling out the forms.

The interventions performed in the present study are described in full detail in a previous publication [12].

### Measures

Hospital Anxiety and Depression Scale (HADS) – This instrument is a widely used tool in screening for anxiety and depressive symptoms in cancer patients [15]. It comprises 14 items distributed across two subscales (HADS-A, anxiety; HADS-D, depression) with seven items each, both ranging from 0 (minimum) to 21 (maximum). It has been validated in Brazil [16]. In the present study, its Cronbach’s alpha ranged from 0.77 to 0.91.

Patient Health Questionnaire (PHQ-9) – The PHQ-9 is considered a very useful instrument for screening for depressive symptoms [17]. It has been previously validated for use in Brazil [18]. It comprises nine questions that investigate symptoms of major depressive episode (DSM-IV), with answers on a 4-point Likert scale (0 to 3) with a maximum score of 27 [17, 18]. In the present study, its Cronbach’s alpha ranged from 0.76 to 0.84.

Edmonton Symptom Assessment System (ESAS) – This instrument consists of a visual numeric scale ranging from 0 to 10 that assesses ten common symptoms in the past 24 h [19]. It was adequately validated for use in Brazil [20]. In the present study, only the “depression” and “anxiety” symptoms and the emotional domain (sum of the scores for depression and anxiety) were considered.

European Organization for Research and Treatment of Cancer Quality of Life Questionnaire Core 15 Palliative (EORTC QLQ-C15-Pal) – This instrument is an abridged version of the EORTC-QLQ-C30 specifically formulated for application to patients with advanced cancer [21]. It comprises 15 items, of which 14 are responded to on a 4-point Likert scale (1 to 4) and one item, which assesses global QOL, is assessed on a numeric scale ranging from 1 to 7. In the present study, only the emotional domain and global QOL were considered. The EORTC-QLQ-C15-Pal was previously validated for use in Brazil [22]. In the present study, its Cronbach’s alpha ranged from 0.85 to 0.90.

Patients completed the HADS, PHQ-9, ESAS, and EORTC QLQ-C15-Pal at days 45, 90, 120 and 180 after randomization; the primary endpoint was previously defined to be measured at 90 days.

### Statistical analysis

The demographic and clinical characteristics before treatment were compared among arms by means of analysis of variance (ANOVA) (continuous variables) and the chi-square test (categorical variables).

The following data were considered for feasibility analysis: the contamination rate (%), calculated as the ratio of the number of patients allocated to arm C who performed at least one consultation with the PC staff over the study period to the total number of patients allocated to arm C; the ratio of included to screened patients (%), calculated as the ratio of the number of patients included in the study to the total number of screened patients; the attrition rate, based on the number of patients lost to follow up for any cause on days 90 and 180.

All scores were compared over time by means of a mixed linear model, considering the treatment group and time-point as fixed effects and patients as the random effect. This technique allowed the effect of the interaction time-point vs. the arm and the effects of the arm and the time-point alone to be investigated, controlling variability by the patient effect. Primary aim was the longitudinal evaluation of depression scores, both measured by HADS-D and PHQ-9.

The difference between time-points 0 and 90 was calculated for each outcome and group, i.e., A, B, and C. Based on these differences, the Cohen’s d effect size (ES) was calculated between arms A and C, B and C, and A and B; it was categorized as small (0.20–0.49), moderate (0.5–0.79), or large (≥0.80) [23]. The difference of means was compared between arms through the Mann-Whitney test with Bonferroni correction; in this case, *p* values ≤0.017 were considered significant.

The intention-to-treat analysis was performed in all cases. The significance level was set to *p* ≤ 0.05. Statistical analysis was performed using the SPSS v.21 software.

### Sample size and planned analysis

Cohen’s effect size was arbitrarily considered moderate to large (Cohen’s d = 0.65) according to decreased HADS-D and PHQ-9 scores when the experimental arms (A or B) were compared with the control group (Arm C). The sample size would be 39 participants in each arm (α = 5%, two-tailed, power = 80%). Taking into account an attrition rate of 25% to 30%, each group shall have a total of 50 patients. An interim analysis was planned after 60 inclusions with 90 days of follow-up to stop the trial (or modify the intervention) in case of effect size <0.2 for the primary study aim (depression scores after 90 days; arms A vs. B).

## Results

**Fig. 1:**
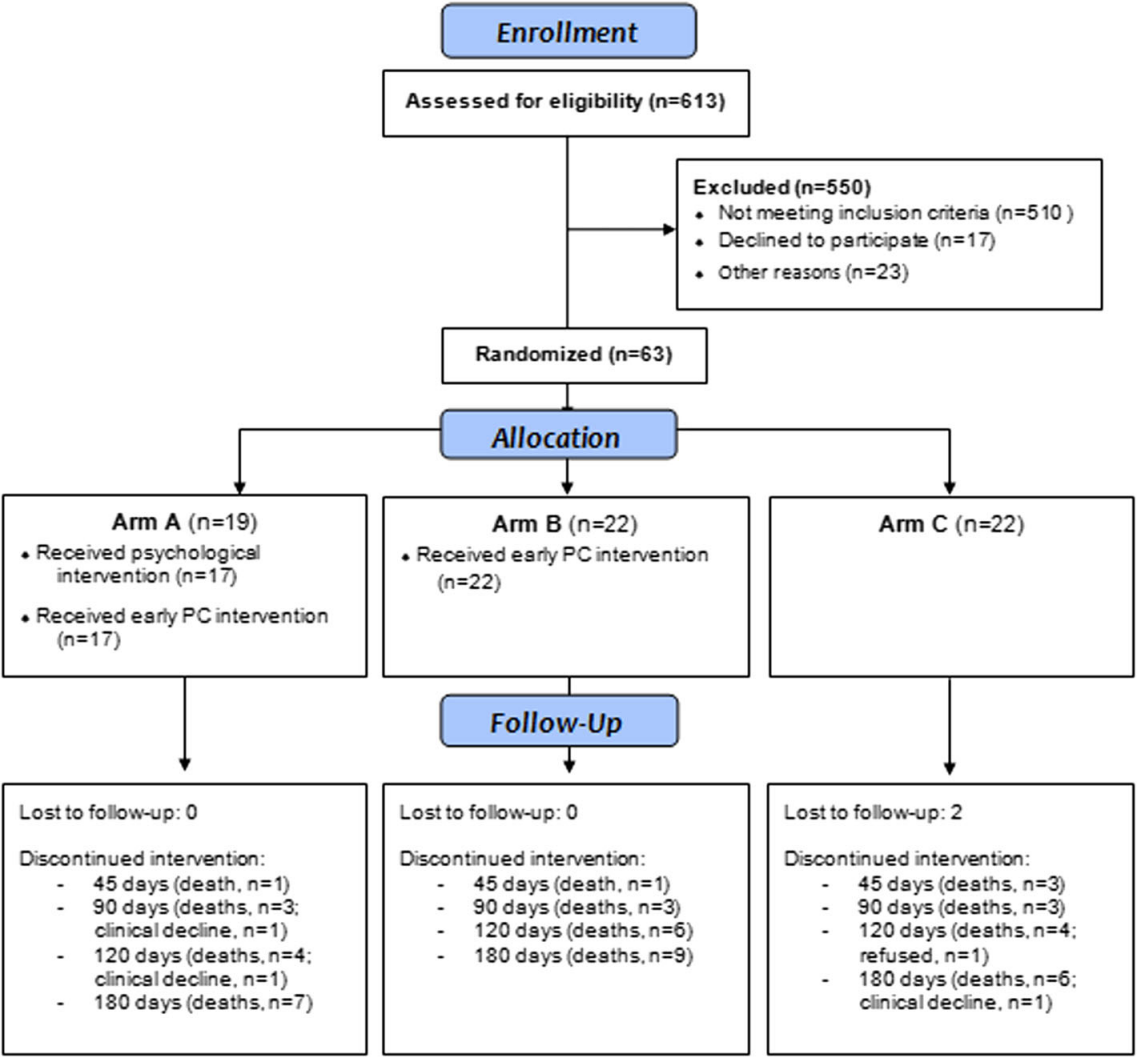
CONSORT diagram showing the flow of participants through the study

Recruitment occurred from August 21, 2014, to August 5, 2015. During this period, 613 patients were screened, 103 were considered potentially eligible, and 63 were finally included (inclusion rate = 10.3%). The main reasons for excluding 510 patients were unavailability to perform the required hospital visits (*n* = 299), use of antidepressants (*n* = 52), age above 75 years old (*n* = 30), diagnosis not compatible with the study criteria (*n* = 26), and life expectancy not compatible with the one required for inclusion (*n* = 23). The main reasons for refusing participation (*n* = 17) were the presence of a limiting comorbidity (*n* = 5) and psychological treatment (*n* = 4) (Fig. 1). Figure 1 depicts the flowchart of patients through the study according to CONSORT and Table 1 describes the sociodemographic characteristics of the sample.

**Table 1:** Baseline characteristics of the participants (*n* = 63)

	Arm A (*n* = 19)	Arm B (*n* = 22)	Arm C (*n* = 22)
Age, mean (SD), years	49.1 (11.1)	52.7 (10.2)	57.0 (11.8)
Female, n (%)	13 (68.4)	14 (62.6)	14 (63.6)
Marital status, n (%)			
Single	4 (21.1)	5 (22.7)	0 (0)
Married	12 (63.2)	11 (50.0)	14 (63.6)
Stable union	1 (5.3)	2 (9.1)	2 (9.1)
Widower	0 (0)	0 (0)	2 (9.1)
Divorced	2 (10.5)	3 (13.6)	2 (9.1)
Other	0 (0)	1 (4.5)	2 (9.1)
Ethnicity, n (%)			
White	12 (63.2)	10 (45.5)	15 (68.2)
Black	3 (15.8)	8 (36.4)	2 (9.1)
Asian	0 (0)	3 (13.6)	1 (4.5)
Brown skin	4 (21.1)	1 (4.5)	4 (18.2)
Religion, n (%)			
Catholic	6 (31.6)	15 (68.2)	15 (68.2)
Evangelical	10 (52.6)	6 (27.3)	6 (27.3)
Jehovah’s Witness	1 (5.3)	1 (4.5)	0 (0)
Kardecist spiritualism	2 (10.5)	0 (0)	1 (4.5)
Low educational level^a^, n (%)			
Low	11 (57.9)	10 (45.5)	12 (54.5)
Professional status, n (%)			
Active	8 (42.1)	8 (36.4)	12 (54.5)
Diagnosis, n (%)			
Breast	6 (31.6)	6 (27.3)	6 (27.3)
Colon and rectum	4 (21.1)	1 (4.5)	2 (9.1)
Prostate	0 (0)	1 (4.5)	1(4.5)
Lung	2 (10.5)	2 (9.1)	3 (13.6)
Head and neck	1 (5.3)	2 (9.1)	1 (4.5)
Cervix	2 (10.5)	4 (18.2)	3 (13.6)
Stomach	1 (5.3)	3 (13.6)	2 (9.1)
Other	3 (15.8)	3 (13.6)	4 (18.2)
HADS-A, mean (SD)	5.79 (3.92)	7.14 (3.41)	6.27 (4.84)
HADS-D, mean (SD)	5.11 (4.51)	7.14 (4.12)	7.18 (4.92)
PHQ-9, mean (SD)	9.7 (6.5)	10.3 (6.0)	9.8 (6.5)
EORTC / Global health, mean (SD)	76.3 (17.0)	72.7 (25.5)	66.7 (29.5)
EORTC / emotional functioning, mean (SD)	70.2 (32.8)	73.5 (21.6)	76.5 (30.7)

Legend: *SD* standard deviation. ^a^Illiterate / reads and writes / incomplete elementary

First, the data were analyzed to establish whether the patients’ characteristics were homogenous across the three arms at baseline; no clinical variable (tumor type, active neoplasm site, educational level, and family income) exhibited differences before intervention, nor did the scores on any of the applied questionnaires (Table 1).

Four patients (arm A, *n* = 2; Arm B, *n* = 2) were diagnosed by clinical oncologists with a depressive disorder during the study and started taking antidepressant (sertraline, *n* = 3; citalopram, *n* = 1). Of these, none were referred for specialized psychiatric treatment.

### Study feasibility

Brief psychosocial intervention *–*Of the 19 patients included in arm A, 15 (78.9%) performed all five sessions and 16 (84.2%) three sessions. One patient (1/19, 5.2%) refused participation in the intervention from the start.

Early palliative care – Of the 41 participants allocated to arms A and B, 22 (53.6%) and 26 (63.4%) performed all the seven and at least 5 (~70% of the scheduled visits) of the scheduled visits to the CP service. It is worth observing that two (4.8%) participants did not attend any visit because they had died before the first scheduled visit. All of the participants were seen by PC staff physicians enrolled to participate in the study; the physicians completed an ad hoc standardized form.

Contamination rate – Of the 22 participants allocated to arm C, only one (4.5%) was seen by the hospital psychology staff during the study period (180 days). In addition, seven (31.8%) were seen by the PC staff over the same period. Therefore, the “contamination” rate over the study period (180 days) was 31.8%.

Losses to follow up – Concerning the assessment on day 90, 10 patients (10/63, 15.9%) were not evaluated, nine due to death and one due to significant worsening of the clinical condition. Regarding the assessment on day 180, the rate of losses was 38% (24/63; death = 22, poor clinical condition = 1, lost of follow-up = 1).

### Longitudinal assessment

**Fig. 2:**
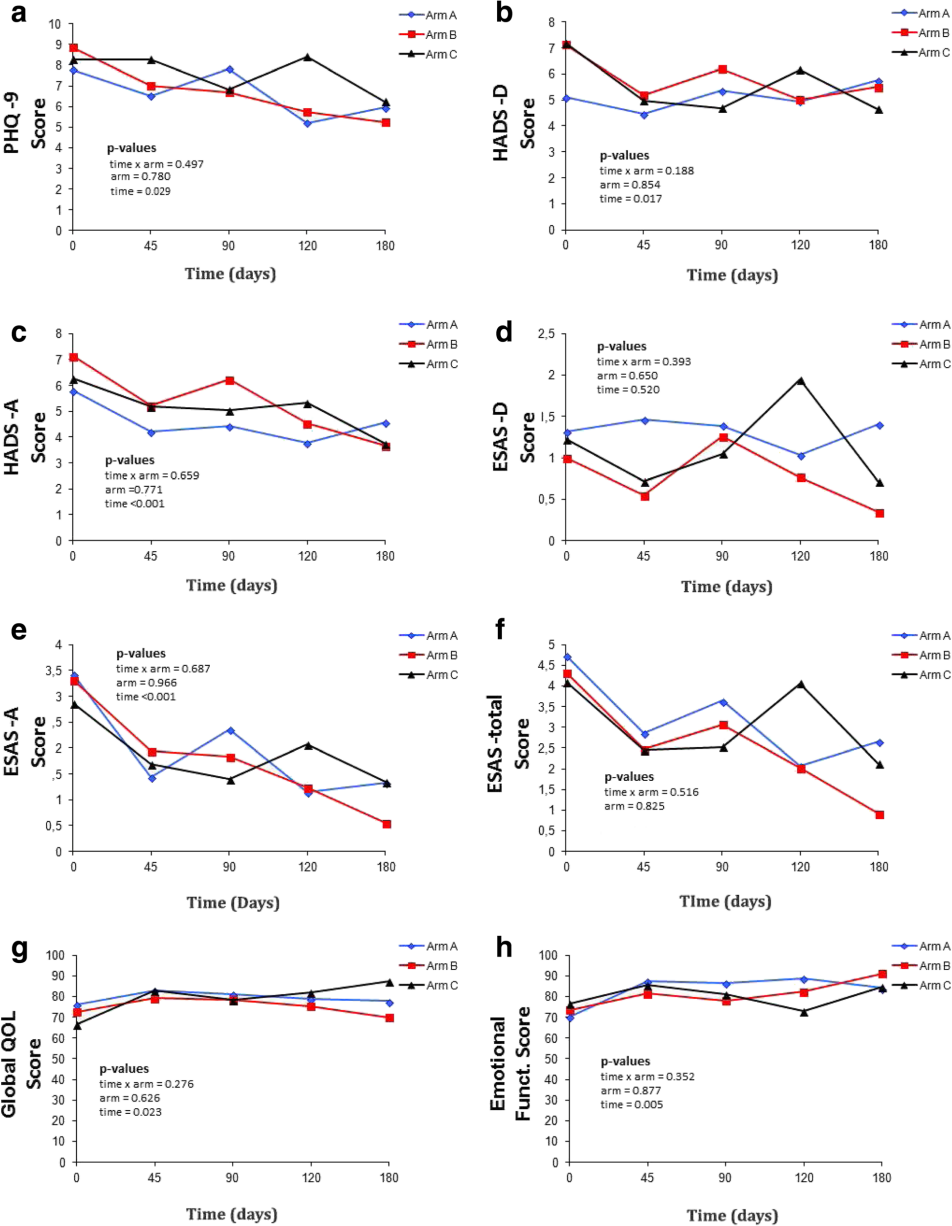
Graphical longitudinal evaluation of the scores on baseline, days 45, 90, 120 and 180 according with the treatment arms. **a**: PHQ-9; **b**: HADS-depression; **c**: HADS-anxiety; **d**: ESAS-depression; **e**: ESAS-anxiety; **f**: ESAS-emotional; **g**: global health (quality of life); **h**: Emotional functioning. Blue line, arm A; red line, arm B; black line, arm C

The scores on the instruments used were assessed over time through comparison using a mixed linear regression model. The significant values of the targeted interaction (arm vs. time-point), arm, and time-point were analyzed. Only ESAS-depression did not exhibit significant differences over time; all other outcomes improved over time. No arm vs. time-point interaction was detected for any of the assessed outcomes (Table 2; Figure 2).

**Table 2:** Difference of means over time among study arms

		A		B		C		*p*-values		
		n	Mean observed change from baseline	n	Mean observed change from baseline	n	Mean observed change from baseline	Time vs. arm	Arm	Time
PHQ-9	d45	18	1.17 (5.71)	21	1.9 (5.03)	19	−0.53 (4.44)	0.497	0.780	0.029
	d90	14	0.21 (4.89)	19	1.95 (5.86)	18	0.94 (4.12)			
	d120	14	2.86 (6.7)	16	3.12 (6.09)	17	−0.76 (5.87)			
	d180	12	2 (7.59)	12	3.83 (4)	14	2.07 (3.69)			
HADS-Anxiety	d45	18	1.56 (3.09)	21	1.9 (3.91)	19	0.89 (4.37)	0.659	0.771	<0.001
	d90	14	1.64 (4.05)	19	1 (4.88)	17	1.24 (5.66)			
	d120	13	2.31 (2.93)	16	2.87 (3.26)	16	1.13 (5.12)			
	d180	12	1.25 (3.89)	11	3.18 (2.79)	14	3.50 (3.16)			
HADS-Depression	d45	17	0.65 (3.12)	21	2.05 (3.73)	19	2.11 (3.73)	0.188	0.854	0.017
	d90	14	−0.43 (3.41)	19	1.11 (5.02)	18	2.44 (3.65)			
	d120	14	0 (2.69)	16	2.69 (4.74)	17	1.12 (4.77)			
	d180	12	−1.08 (4.46)	12	1.83 (4.06)	14	2.79 (4.04)			
ESAS- Anxiety	d45	18	2.11 (3.5)	21	1.48 (2.52)	19	0.95 (3.42)	0.687	0.966	<0.001
	d90	14	1.71 (2.46)	19	1.58 (3.95)	18	1.17 (3.33)			
	d120	14	2.93 (3.17)	16	2.13 (2.68)	17	0.59 (4.06)			
	d180	12	2.92 (3.20)	12	2.42 (2.43)	14	1.36 (3.20)			
ESAS-Depression	d45	18	0.11 (3.01)	21	0.48 (1.89)	19	0.37 (1.3)	0.393	0.650	0.520
	d90	14	0.21 (3.75)	19	0.42 (1.68)	18	0.06 (2.88)			
	d120	14	0.57 (2.31)	16	0.25 (1.73)	17	−0.82 (3)			
	d180	12	0.25 (2.09)	12	0.42 (1)	14	0.43 (1.91)			
ESAS- Emotional domain	d45	18	2 (4.17)	21	1.95 (3.07)	19	1.32 (4.57)	0.516	0.825	0.001
	d90	14	1.93 (5)	19	1.16 (4.54)	18	1.22 (5.77)			
	d120	14	3.5 (3.98)	16	2.38 (3.59)	17	0.24 (6.69)			
	d180	12	3.17 (2.55)	12	2.83 (2.72)	14	1.79 (4.69)			
EORTC QLQC-15Pal Total	d45	17	−5.88 (18.58)	21	−7.14 (24.48)	19	−15.79 (32.62)	0.276	0.626	0.023
	d90	14	4.76 (23.96)	19	−7.02 (16.02)	18	−12.04 (21.24)			
	d120	14	−2.38 (22.51)	16	−4.17 (28.87)	17	−16.67 (30.05)			
	d180	12	0 (22.47)	12	2.78 (35.41)	14	−20.24 (27.09)			
EORTC QLQC-15Pal Emotional domain	d45	18	−17.59 (29.96)	21	−8.73 (21.49)	19	−7.89 (28.53)	0.352	0.877	0.005
	d90	14	−20.24 (29.37)	19	−3.51 (21.93)	18	−3.70 (26.54)			
	d120	14	−22.62 (26.64)	16	−8.33 (29.81)	17	3.92 (36.1)			
	d180	12	−16.67 (30.15)	12	−15.28 (15.01)	14	−9.52 (38.52)

### Assessment on day 90

Regarding depressive symptoms, analysis of the ES showed that patients subjected to the experimental intervention (arm A) worsened compared to arms B and C. In turn, arm A had better results in emotional functioning compared to arms B (ES = −0.66) and C (ES = −0.61). Assessment of the difference between means with the non-parametric Mann-Whitney test showed a *p*-value <0.05 for the HADS-depression domain. However, following Bonferroni correction, this difference was not significant (*p* > 0.017; Table 3).

**Table 3:** Difference in means and effect size among study arms

Instrument	Assessed domain	Comparison among study arms											
		A vs. B				A vs. C				B vs. C			
		Δ A	Δ B	ES	p*	Δ A	Δ C	ES	p*	Δ B	Δ C	ES	p*
EORTC QLQ-C15 Pal	Global health	−4.76	−8.88	0.20	0.953	−4.76	−11.76	0.31	0.984	−8.88	−11.76	0.15	0.946
	Emotional functioning	−20.24	−2.94	−0.66	0.200	−20.24	−2.94	−0.61	0.138	−2.94	−2.94	0.00	0.734
HADS	Anxiety	1.64	0.82	0.17	0.984	1.64	0.88	0.15	0.951	0.82	0.88	−0.01	0.958
	Depression	−0.43	1.06	−0.33	0.336	−0.43	2.18	−0.74	0.029	1.06	2.18	−0.25	0.394
PHQ9	Depression	1.14	2.71	−0.26	0.356	1.14	0.88	0.05	0.710	2.71	0.88	0.32	0.231
ESAS	Anxiety	1.93	0.88	0.22	0.710	1.93	0.88	0.19	0.173	0.88	0.88	0.00	0.290
	Depression												
	Emotional	1.93	0.88	0.22	0.710	1.93	0.88	0.19	0.173	0.88	0.88	0.00	0.290

Legend: *ES* effect size; Δ = difference in means between days 0 and 90 (d0-d90)

*Mann-Whitney test (Bonferroni correction; values are significant when *p* < 0.017)

## Discussion

In this study, we assessed the feasibility and the efficacy of a brief psychosocial intervention systematically combined with early PC to reduce symptoms of depression 90 days after randomization. To our knowledge, no previous study has tested a brief psychosocial intervention together with early PC in the same clinical context. Depressive symptoms did not significantly differ between the arm that received psychosocial intervention and early PC and the other two arms over time.

Scientific evidence demonstrates the benefits of cognitive-behavioral approaches in the reduction of depressive symptoms among cancer patients [24]. A previous study demonstrated a reduction in anxiety and an improvement in QOL scores among end-stage cancer patients receiving CBT. The authors stressed the relevance of fitting interventions to the needs of this particular population of patients [25]. A large amount of scientific evidence demonstrates the efficacy of the CBT approach in cancer patients, including improvement in the state of health, changes in behavior, reduction in significant symptoms and depression, and greater independence in symptom management [26–28]. However, there are no reports of studies that use CBT together with early PC in patients with advanced cancer, which we consider to be a population at high risk for the development of depression and anxiety.

Because cancer is a chronic, incurable disease with a substantial impact on patients’ life routine, family functioning, roles performed, physical functioning, and professional activities, among other areas, patients will expectably exhibit some psychological maladjustment. Some symptoms, such as those of depression and anxiety, are easily found under these circumstances [29, 30]. Some authors stress the need to investigate depression at different times to identify a potential healthy adjustment associated with treatment [31]. In the present study, we found that both depression and anxiety symptoms improved over time, independent of the interventions received; thus, one may expect such symptoms to naturally decrease over the course of treatment. It should be emphasized that the participants were at the point of starting first-line chemotherapy, i.e., they had already been informed as to the occurrence of relapse or progression of the disease, which most likely elicited higher levels of emotional symptoms. In addition, the feeling/fear of the unknown is frequent at the onset of chemotherapy (“Will I feel sick?”, “How will I feel after treatment?”, etc.). We believe that there is a trend for the improvements in depression and anxiety levels with the progression of treatment.

The tendency of patients subjected to the psychosocial intervention to exhibit higher scores of depression over time compared to the other two arms was a significant and unexpected finding. According to some studies, debriefing, i.e., talking about some traumatic event immediately after it occurs, may intensify uncomfortable symptoms [32, 33]. In this regard, one should consider the fact that a diagnosis of cancer is related to a significantly traumatic event [34], particularly in the case of advanced and incurable disease. On these grounds, the time of application of the psychosocial intervention, i.e., immediately after diagnosis of cancer relapse/progression, may have worsened some emotional aspects. Future studies including psychological strategies need to consider what the most adequate time for intervention may be. Probably, the most useful protocol would include on-demand psychological intervention, but not systematic as was in our study. However, regarding the emotional functioning domain, arm A responded better than arms B and C. The same held true for anxiety symptoms, albeit with a smaller effect sizes.

We consider the preliminary results of this study very important for the design of a larger multicenter, multinational randomized clinical trial. Regarding feasibility, some aspects warrant discussion. The eligibility criteria are being revised to become less restrictive, since the inclusion rate was only 10.2%. Among other modifications in the research protocol, we plan not to limit age at 75 years, and not to exclude patients because of previous psychiatric conditions. Additionally, aiming to minimize the burden of completing many questionnaires and considering that PHQ-9 and HADS measures a similar construct (depression), we plan to use only HADS in the larger study. Another factor that limited the study feasibility was the high contamination rate found; approximately 30% of the patients allocated to arm C received PC during the study period. This value is slightly more than twice the rate reported in the original study by Temel et al. [3]. However, we think that the intention-to-treat analysis used likely reduced the contamination bias. The attrition rate was 15.9% and 38% on days 90 and 180, respectively. Although these rates may be considered high, they are similar (and actually somewhat lower) than those previously described [35]. One of the goals of early PC is to help patients navigate the difficult decisions made towards the end of life which would include the decision whether or not to undergo palliative chemotherapy. In order to enhance the benefit of PC, in the subsequent study, we plan to include patients before the decision to receive first-line palliative chemotherapy, and not after starting chemotherapy (as in the present study).

The present study has several limitations, including the fact that it was a single-center study. The study was performed at a hospital that provides adequate psychosocial support as “routine”; thus, findings may be different in less specialized hospitals. Another limitation is the sample size. The study did not reach its enrollment goal and is likely underpowered to evaluate its primary outcome. In addition, due to the restricted eligibility criteria, the findings are difficult to generalize. The focus was on the prevention of depressive symptoms. For this reason, patients with a diagnosis of depression or known to be using antidepressants were excluded. As a result, the depression scores most likely may have been lower than those found in daily clinical practice, leaving little “room” for improvement. In addition, a slight worsening of the clinical condition may be clinically relevant. This fact may be explained by the statistical phenomenon known as regression to the mean, which has potentially significant implications for the interpretation of health-related behaviors [36]. Further studies should focus on patients reporting higher scores of anxiety and depression.

## Conclusions

Future studies to be conducted with this population group need to revise the eligibility criteria and make them less restrictive. In addition, the need for arm C is questioned due to high contamination rate. The systematic psychosocial and educational intervention was not able to reduce the depression scores after 90 days or over time in patients starting first-line palliative chemotherapy. Assessment of the ES indicates a possible impact of interventions on the emotional functioning domain, which need to be better assessed in future studies. The intervention should be tested on-demand and in subgroups of high risk of anxiety and depression.

## Additional file

Additional file 1: Detailed structured psychosocial and educational intervention. Description of the structured psychosocial and educational intervention used in the study. (PDF 129 kb)

## Abbreviations

ANOVA: Analysis of variance
BCH: Barretos Cancer Hospital
CBT: Cognitive-behavioral therapy
ECOG-PS: Eastern Cooperative Oncology Group
EORTC-QLQ-C15-Pal: European Organization for Research and Treatment of Cancer Quality of Life Questionnaire Core 15 Palliative
ES: Effect size
ESAS: Edmonton Symptom Assessment System
HADS: Hospital Anxiety and Depression Scale
PC: Palliative care
PHQ-9: Patient Health Questionnaire
PREPArE: Pre-Palliative Emotional Care
QOL: Quality of life

## References

[1] LeBlanc TW, Nickolich MS, El-Jawahri A, Temel JS. Discussing the Evidence for Upstream Palliative Care in Improving Outcomes in Advanced Cancer. Am. Soc. Clin. Oncol. Educ. B. [Internet]. 2016 [cited 2016 Nov 29];36:e534–8. 10.1200/EDBK_159224 27249764

[2] Smith TJ, Temin S, Alesi ER, Abernethy AP, Balboni TA, Basch EM (2012). American Society of Clinical Oncology provisional clinical opinion: the integration of palliative care into standard oncology care. J Clin Oncol.

[3] Temel JS, Greer JA, Muzikansky A, Gallagher ER, Admane S, Jackson VA (2010). Early palliative care for patients with metastatic non-small-cell lung cancer. N Engl J Med.

[4] Zimmermann C, Swami N, Krzyzanowska M, Hannon B, Leighl N, Oza A (2014). Early palliative care for patients with advanced cancer: a cluster-randomised controlled trial. Lancet.

[5] Bakitas M, Lyons KD, Hegel MT, Balan S, Brokaw FC, Seville J (2009). Effects of a palliative care intervention on clinical outcomes in patients with advanced cancer: the Project ENABLE II randomized controlled trial. JAMA.

[6] Maltoni M, Scarpi E, Dall’Agata M, Schiavon S, Biasini C, Codecà C (2016). Systematic versus on-demand early palliative care: A randomised clinical trial assessing quality of care and treatment aggressiveness near the end of life. Eur J Cancer.

[7] McDonald J, Swami N, Hannon B, Lo C, Pope A, Oza A, et al. Impact of early Palliative Care on Caregivers of Patients with Advanced Cancer: Cluster Randomised Trial. Ann Oncol. 2016; 10.1093/annonc/mdw438. 10.1093/annonc/mdw438 27687308

[8] Hui D, Kim S-H, Kwon JH, Tanco KC, Zhang T, Kang JH (2012). Access to palliative care among patients treated at a comprehensive cancer center. Oncologist.

[9] Miyashita M, Hirai K, Morita T, Sanjo M, Uchitomi Y (2008). Barriers to referral to inpatient palliative care units in Japan: a qualitative survey with content analysis. Support Care Cancer.

[10] Gott M, Ingleton C, Bennett MI, Gardiner C (2011). Transitions to palliative care in acute hospitals in England: qualitative study. BMJ.

[11] Rugno FC, Paiva BSR, Nunes JS, Paiva CE (2014). “There won’t' be anything else...it's over”: Perceptions of women referred to palliative care only. Eur J Oncol Nurs.

[12] Do Carmo TM, Paiva BSR, de Siqueira MR, L de TB d R, de Oliveira CZ, de A Nascimento MS (2015). A phase II study in advanced cancer patients to evaluate the early transition to palliative care (the PREPArE trial): protocol study for a randomized controlled trial. Trials.

[13] Pitceathly C, Maguire P, Fletcher I, Parle M, Tomenson B, Creed F (2009). Can a brief psychological intervention prevent anxiety or depressive disorders in cancer patients? A randomised controlled trial. Ann Oncol.

[14] Beck J (2011). Cognitive Behavior Therapy: Basics and Beyond.

[15] Zigmond AS, Snaith RP (1983). The hospital anxiety and depression scale. Acta Psychiatr Scand.

[16] Botega NJ, Bio MR, Zomignani MA, Garcia C, Pereira WA (1995). Mood disorders among inpatients in ambulatory and validation of the anxiety and depression scale HAD. Rev Saude Publica.

[17] Kroenke K, Spitzer RL, Williams JB (2001). The PHQ-9: validity of a brief depression severity measure. J Gen Intern Med.

[18] Santos IS, Tavares BF, Munhoz TN, de ALSP, da SNTB, Tams BD (2013). Sensitivity and specificity of the Patient Health Questionnaire-9 (PHQ-9) among adults from the general population. Cad Saude Publica.

[19] Bruera E, Kuehn N, Miller MJ, Selmser P, Macmillan K (1991). The Edmonton Symptom Assessment System (ESAS): a simple method for the assessment of palliative care patients. J Palliat Care.

[20] Paiva CE, Manfredini LL, Paiva BSR, Hui D, Bruera E (2015). The Brazilian Version of the Edmonton Symptom Assessment System (ESAS) Is a Feasible, Valid and Reliable Instrument for the Measurement of Symptoms in Advanced Cancer Patients. PLoS One.

[21] Groenvold M, Petersen MA, Aaronson NK, Arraras JI, Blazeby JM, Bottomley A (2006). The development of the EORTC QLQ-C15-PAL: a shortened questionnaire for cancer patients in palliative care. Eur J Cancer.

[22] Nunes NAH (2014). The quality of life of Brazilian patients in palliative care: validation of the European Organization for Research and Treatment of Cancer Quality of Life Questionnaire Core 15 PAL (EORTC QLQ-C15-PAL). Support Care Cancer.

[23] Cohen J (1988). Statistical Power Analysis for the Behavioral Sciences.

[24] Sherwood P, Given BA, Given CW, Champion VL, Doorenbos AZ, Azzouz F (2005). A cognitive behavioral intervention for symptom management in patients with advanced cancer. Oncol Nurs Forum.

[25] Greer JA, Traeger L, Bemis H, Solis J, Hendriksen ES, Park ER (2012). A pilot randomized controlled trial of brief cognitive-behavioral therapy for anxiety in patients with terminal cancer. Oncologist.

[26] Dobson KS, Dozois DJA (2006). Fundamentos históricos e filosóficos das terapias cognitivo-comportamentais. Manual de Terapias Cognitivo-Comportamentais.

[27] Quesnel C, Savard J, Simard S, Ivers H, Morin CM (2003). Efficacy of cognitive-behavioral therapy for insomnia in women treated for nonmetastatic breast cancer. J Consult Clin Psychol.

[28] Lovejoy NC, Tabor D, Matteis M, Lillis P (2000). Cancer-related depression: Part I--Neurologic alterations and cognitive-behavioral therapy. Oncol Nurs Forum.

[29] Krebber AMH, Buffart LM, Kleijn G, Riepma IC, de Bree R, Leemans CR (2014). Prevalence of depression in cancer patients: a meta-analysis of diagnostic interviews and self-report instruments. Psychooncology.

[30] Lima MP, Longatto-Filho A, Osório FL (2016). Predictor Variables and Screening Protocol for Depressive and Anxiety Disorders in Cancer Outpatients. PLoS One.

[31] Lo C, Zimmermann C, Rydall A, Walsh A, Jones JM, Moore MJ (2010). Longitudinal Study of Depressive Symptoms in Patients With Metastatic Gastrointestinal and Lung Cancer. J Clin Oncol.

[32] Jacobs AK, Pfefferbaum B (2015). The Use of Debriefing With Children. Curr Psychiatry Rep.

[33] Ehlers A, Clark D (2003). Early psychological interventions for adult survivors of trauma: a review. Biol Psychiatry.

[34] Swartzman S, Booth JN, Munro A, Sani F. Posttraumatic stress disorder after cancer diagnosis in adults: A meta-analysis. Depress Anxiety. 2016; 10.1002/da.22542. 10.1002/da.22542 27466972

[35] Hui D, Glitza I, Chisholm G, Yennu S, Bruera E (2013). Attrition rates, reasons, and predictive factors in supportive care and palliative oncology clinical trials. Cancer.

[36] Linden A (2013). Assessing regression to the mean effects in health care initiatives. BMC Med Res Methodol.

