# Supplementary material for: The feasibility and benefit of a brief psychosocial intervention in addition to early palliative care in patients with advanced cancer to reduce depressive symptoms: a pilot randomized controlled clinical trial
Source: BMC Cancer. 2017 Aug 23;17:564. doi: 10.1186/s12885-017-3560-6 (PMC5569457; doi:10.1186/s12885-017-3560-6)
Supplement: Additional file 1: — Detailed structured psychosocial and educational intervention. Description of the structured psychosocial and educational intervention used in the study. (PDF 129 kb) [file 12885_2017_3560_MOESM1_ESM.pdf]

Supplementary Table 1. Detailed structured psychosocial and educational intervention

Week	Planned activities
1	• Introduction of the therapist/patient and establishment of rapport<br>• Objective measurement of depression and anxiety symptoms<br>• Psychoeducation: disease - explanation of the cancer diagnosis and current health status; symptoms - a discussion of uncomfortable symptoms secondary to the cancer; adverse events - an explanation of adverse events that result from treatment procedures; PC - What is it? What is it for? For who is it provided? When is it provided? How is it provided? Where is it provided?<br>• Delivery of the "Educational Booklet" that was developed specifically for this study[1]<br>• Establishment of therapeutic goals<br>• "Partnership Agreement" – the moment when the therapist and the patient sign a commitment to walk together on the path that will be traveled by both during the five proposed sessions<br>• Establishment of the dates and times of the sessions<br>• Session summary and feedback
2	• Assessment of weekly progress and a bridge to the previous session<br>• Objective measurement of depression and anxiety symptoms<br>• Reinforcement of the therapeutic goals that were set in the previous session<br>• Early PC – description of the benefits of simultaneous monitoring by a PC team for advanced cancer patients who are submitted to palliative chemotherapy and presentation of the explanatory figure "Goals of Care"<br>• Delivery of the Coping Card[1]<br>• Session summary and feedback
3	• Assessment of weekly progress and a bridge to the previous session

	• Objective measurement of depression and anxiety symptoms<br>• Anxiety mechanisms: perceived symptoms and how to monitor these symptoms<br>• Relaxing training - image association<br>• Session summary and feedback
4	• Assessment of weekly progress and a bridge to the previous session<br>• Objective measurement of depression and anxiety symptoms<br>• Mechanisms of depressive symptoms: perceived symptoms and how to monitor these symptoms<br>• Identification of automatic thoughts (thought > emotion > behavior), explanation of how thoughts affect emotions and therefore behaviors, and elucidating the key role of thought on emotion and behavior<br>• Patient preparation for discontinuation following the next session<br>• Delivery of the Coping Card[1]<br>• Session summary and feedback
5	• Assessment of weekly progress<br>• Objective measurement of depression and anxiety symptoms<br>• Retrospective of the methods used in the previous sessions, including progress, difficulties, commitment, and learning<br>• Discussion of the patient’s ability to control and understand the process as a way to prevent serious symptoms<br>• General feedback

[1]The Educational Booklet contains practical information in layman’s terms about the possible symptoms secondary to chemotherapy treatment and symptoms that are often associated with advanced cancer. This booklet explains the importance of the clinical support that is provided by teams that specialize in palliative care.

[2]The Coping Cards were prepared using stimulating sentences, and guidelines will be provided during the sessions regarding the appropriate moments when the cards should be read or remembered.
